# Increased Signal Complexity Improves the Breadth of Generalization in Auditory Perceptual Learning

**DOI:** 10.1155/2013/879047

**Published:** 2013-11-14

**Authors:** David J. Brown, Michael J. Proulx

**Affiliations:** ^1^Biological and Experimental Psychology Group, School of Biological and Chemical Sciences, Queen Mary University of London, Mile End Road, London E1 4NS, UK; ^2^Department of Psychology, University of Bath, Bath BA2 7AY, UK

## Abstract

Perceptual learning can be specific to a trained stimulus or optimally generalized to novel stimuli with the breadth of generalization being imperative for how we structure perceptual training programs. Adapting an established auditory interval discrimination paradigm to utilise complex signals, we trained human adults on a standard interval for either 2, 4, or 10 days. We then tested the standard, alternate frequency, interval, and stereo input conditions to evaluate the rapidity of specific learning and breadth of generalization over the time course. In comparison with previous research using simple stimuli, the speed of perceptual learning and breadth of generalization were more rapid and greater in magnitude, including novel generalization to an alternate temporal interval within stimulus type. We also investigated the long term maintenance of learning and found that specific and generalized learning was maintained over 3 and 6 months. We discuss these findings regarding stimulus complexity in perceptual learning and how they can inform the development of effective training protocols.

## 1. Introduction

Animals improve in the extraction and encoding of sensory information from the environment through perceptual learning. Psychophysical studies have established that practicing a task leads to specific improvements that are often restricted to stimuli used during training [1, 2].**   **While these paradigms typically utilise simple unisensory stimuli, the reverse hierarchy theory of perceptual learning is consistent with evidence that the “default” setting in perception is one of higher order complex objects. For example, ecologically it is unusual to be presented with simple pure tones in isolation, but rather the complex frequency changes present in vocal communication such as birdsong and human speech [3–5]. 

Auditory research shows that while specific learning is found in most tasks, generalization to novel stimuli is generally restricted to spectral features of the stimuli [6–11].

In contrast, generalization to temporal stimulus features appears to be very limited, although it has been found for transferral from interval to duration within the same stimulus length, and onset/offset asynchrony, respectively [12, 13]. With regard to generalization to new intervals/durations, although Lapid and colleagues reported such generalization [14], this is in contrast with the majority of studies in which no such transfer of learning is found [11, 12, 15] with Lapid's study demonstrating generalization across stimulus types (Empty-Filled). This limitation of generalization appears to be consistent even after extensive training [11, 15, 16], and with spectral feature processing and specific learning attributed to initial regions in the auditory cortex, there is no anatomical limitation to this neural plasticity. However, temporal generalization may be sited in secondary auditory and multisensory areas utilising top-down processes to facilitate this learning. One key might be the use of simple versus complex stimuli during training [17–19]. 

Here we investigated the perceptual learning of complex auditory stimuli. Utilising an established temporal interval discrimination paradigm [11], we tested the specificity of learning to filled complex stimuli and the generalization to untrained durations within the same stimulus type. Using the data from Wright et al. [11] as a comparison for simple stimulus based perceptual learning, we tested whether the use of complex stimuli would speed perceptual learning and increase the breadth of generalization. We adapted the classic auditory learning paradigm in two ways [11, 15, 16]. First, the stimuli were complex, created by sonifying an image using a visual-to-auditory sensory substitution device (SSD) called the vOICe [20]. This device uses cross-modal correspondences to transmit sensory information usually associated with an impaired modality (vision) via an unimpaired modality (audition). From an applied perspective, it aims to give a basic visual percept to the visually impaired community whilst theoretically acting as a valuable tool to evaluate multisensory processes in perception. We created our stimuli using this device because the transformation algorithm of this device ensures that the auditory output signal is necessarily complex as over 4000 sonified “visual” pixels create a soundscape comprised of multiple frequency and temporal components. Moreover, not only has this device been used to investigate the neural basis of auditory object recognition [21, 22] and localization [23] but also results from this experiment could be extrapolated to help formulate effective training paradigms for sensory substitution device usage. The second adaptation to the paradigm used by Wright and colleagues was the use of filled durations, rather than empty intervals in both the training and test phases to evaluate whether the use of within type complex stimuli would facilitate a learning advantage over simple stimuli. The literature has shown that while discrimination differences, have been shown for empty intervals and filled durations, the methodology (2AFC) and durations (90–220 ms) utilised in the present experiment show no significant differences, and therefore comparisons with empty interval paradigms are valid [24].

Based on applying RHT to auditory stimuli, we hypothesized that complex stimuli can be learned specifically and also can increase the breadth of generalization. We also predicted that if signal complexity facilitates generalization, then the use of The vOICe's stereo mode, with its two factor principle for horizontal spatial localisation, would outperform the monaural setting. If perceptual learning occurs at a higher, central neural level and results in generalization due to stimulus complexity, it is possible that such neural plasticity should be long lasting [25]. While maintenance of perceptual learning has been demonstrated over 4 and 8 weeks, respectively, [6, 26] we extended this time frame by conducting a follow-up experiment after 3 and 6 month periods signified by an absence of further training. 

## 2. Materials and Methods 

### 2.1. Subjects

Twenty-four paid listeners (15 female) were recruited. Listener age range was between 19 and 35 (*m* = 23.50,   S.D. = 4.9). All listeners reported normal hearing, normal or corrected eyesight, a formal education to undergraduate level or above, and a good understanding of the English language and provided written informed consent. Twenty-one of the listeners were self-reported as right handed. Listeners were assigned to experimental groups in a pseudorandom manner aside from the gender split, where 5 females were in each group. Each group completed the same task but was differentiated on the number of training days undertaken (2, 4, or 10).

### 2.2. Materials

Stimuli were designed using The vOICe [20] and Adobe Audition 3—see “Stimulus Design”. The script was run in Matlab and Psychtoolbox [27–29] on a Windows PC with a Creative Labs Soundblaster Titanium ASIO soundcard to ensure low latency. All auditory signals were transmitted to the listener through Sennheiser HD555 noise reducing over ear headphones. The blindfold used was the Mindfold Inc. (Tucson, AZ).

### 2.3. Stimulus Design

A plain white triangle (apex upwards) on a black background was sonified using The vOICe's image sonification feature. Prior to sonification, the device scan rate was set at “×8” to reduce the temporal length of the stimulus to 125 milliseconds. This was then trimmed to remove the soundscape representing the black areas at each side of the triangle base resulting in an auditory stimulus of 90 ms. Adobe Audition 3 was used to apply a 10 ms cosine ramp fade in and out to the stimulus onset and offset. Frequency was measured as a range, as the experimental aim was to create a complex signal comprised of a range of frequencies (each of the 4096 pixels has its own frequency, amplitude and temporal feature). For the standard stimulus, the fundamental frequency was centered at 1 kilohertz (kHz), temporal interval of 90 ms, and amplitude of −85 dB. The alternate “test” stimuli were created by manipulating the standard stimulus in either Adobe Audition 3 (frequency) or The vOICe (duration). The frequency range was increased using a 0.60 ratio that raised the frequency range to one centered at 4 kHz whilst retaining the 90 ms temporal interval. The alternate duration was generated using the same visual stimulus but sonified using The vOICe at a 250 ms scan rate. After the trim and ramp were applied, the resultant stimuli were at 1 khz frequency with a temporal interval of 220 ms. For the stereo condition the frequency and duration values were the same as the standard (90 ms, 1 kHz), but the signal was conveyed through both headphones binaurally. 

**Figure 1:**
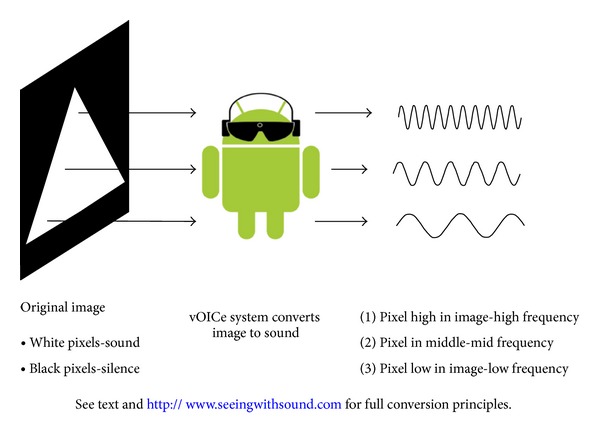
Conversion of image to sound using The vOICe algorithm. White pixels in the image are represented by a sound with black pixels silent. The elevation of each pixel is coded by frequency with pixels higher in the image having a higher frequency sine wave. All pixels in a vertical raster line are played simultaneously with a 1 second left-to-right horizontal scan across image resulting in the soundscape for the image.

Figure 1 shows how The vOICe sonifies visual images in real time converting visual features (brightness and spatial position) to auditory features (amplitude, frequency, time, and stereo panning). Each of the 4096 pixels in the recorded greyscale image is subjected to 3 conversion principles. Visual brightness is coded to auditory amplitude with brighter pixels eliciting louder tones. Spatial position uses two principles to code for vertical and horizontal localisation. On the  *y-*axis pixel position corresponds to frequency with higher frequencies representing pixels higher up in the recorded image. A one second left-to-right time scan across the image provides a temporal cue to position on the  *x-*axis with pixels to the left of the image being heard earlier in the time scan. If used in stereo mode a left-to-right pan across the stereo field provides, in conjunction with the time scan, a more accurate and complex coding feature for horizontal localisation with left orientated pixels being heard in the left headphone. To give the final “soundscape”, all pixel sounds in a column are played concurrently (64 pure tones imposed over each other) with these 174 raster lines then played sequentially over the duration of the time scan. The resulting “soundscape”, is a complex signal comprised of a large number of frequencies and amplitudes, played back to the user either monaurally or binaurally via headphones. 

### 2.4. Procedure

Listeners were assigned a work station; the procedure was explained to them both verbally and via an information sheet, and written consent was obtained. The blindfold and headphones were then put on, and each listener was guided to the “1” and “2” keys on the number pad on the PC keyboard. Listeners were then instructed to press the spacebar twice to start the first block of 60 trials. This double press of the spacebar was used to start all blocks in the condition (9 on training days and 5 for test days).

**Figure 2:**
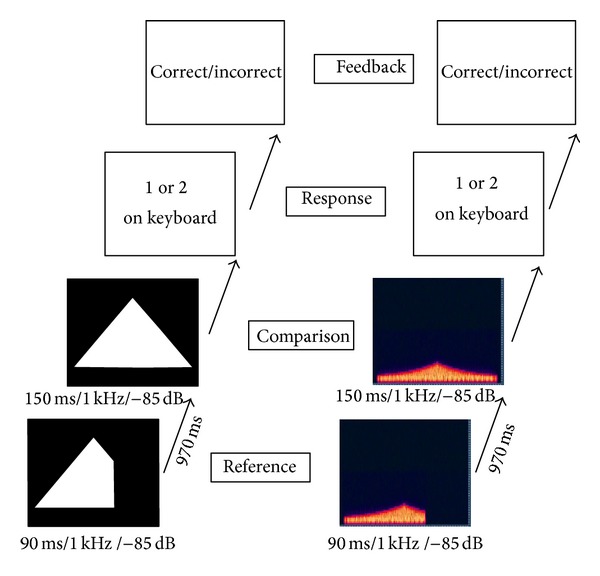
Representation of a sample trial. Listeners are presented with a reference soundscape followed by a 970 ms interstimulus gap. They are then presented with a comparison tone and are required to indicate whether the reference tone was presented 1st or 2nd. In the standard condition the reference tone is always of the same duration, with reference and comparison tones presented in a random order. Feedback is given after the response before the onset of the next trial. The duration of the reference tone is stable with the comparison tone adapted on a 3 up/1 down staircase procedure. The left hand column of the figure shows the image that was sonified, with the right hand column showing the spectrogram for the resultant soundscape.

Figure 2 displays a sample trial for the standard condition. For each trial the listeners were presented with a pair of tones, separated by 970 ms, in the left headphone. One of these tones was the “reference” tone (*t*) which was temporally consistent throughout all trials in the particular condition. The other “comparison” tone (*t* + Δ*t*) varied in duration depending on previous answers and the 3 up/1 down psychophysical staircase procedure. 

Three correct consecutive responses reduced the Δ*t* by 1 unit whilst one incorrect response increased the Δ*t* by one unit. The trial where the direction changed, from decrease to increase or vice versa, was classed as a reversal. For the first three reversals the unit change was 5 ms with a 1 ms change for subsequent reversals in each block.

Listeners were required to indicate using the number keys whether the reference tone was presented first or second in the pairing. After the keystroke was made, feedback was provided by a “pure tone” in the right headphone for an incorrect answer followed by the onset of the next trial. Correct responses resulted in the next trial starting with no prior auditory feedback.

After a 60-trial block was completed, the next block was initiated by the listener by a double depression of the space bar. This allowed the listener to take a short break at their own discretion. “Official” breaks were also offered between the 5th and 6th blocks on a training day. During this intermission, the listeners were allowed to remove the headphones but not the blindfold. On the test days short breaks were taken between the conditions whilst the next conditions, script was loaded into Matlab, and an official break was offered after the first two conditions (10 blocks). The average time duration per block was four minutes.

The pretest consisted of 5 blocks of each of the 4 conditions, standard, alternate interval, alternate frequency, and stereo (1200 trials in total). The presentation of the conditions was varied amongst groups but was kept consistent within group concerning the pre and posttests. The standard condition was presented first for all groups in the pretest phase. 

### 2.5. Calculation of Thresholds

Thresholds were obtained by first removing the first 3 or 4 reversals in each block to ensure an even number of reversals. If this resulted in there being less than 6 reversals in the block then the block was disregarded. For the accepted blocks the  Δ*t*  for each of the reversals was noted and averaged across the block to give a block threshold. On the proviso that there were at least 3 (pre- and posttest) or 6 (training) thresholds, mean scores were calculated for individual listeners and experimental groups for each session. Weber fractions were computed by dividing the total Δ*t* by  *t*  and then entered for analysis.

## 3. Results

### 3.1. Learning

**Figure 3:**
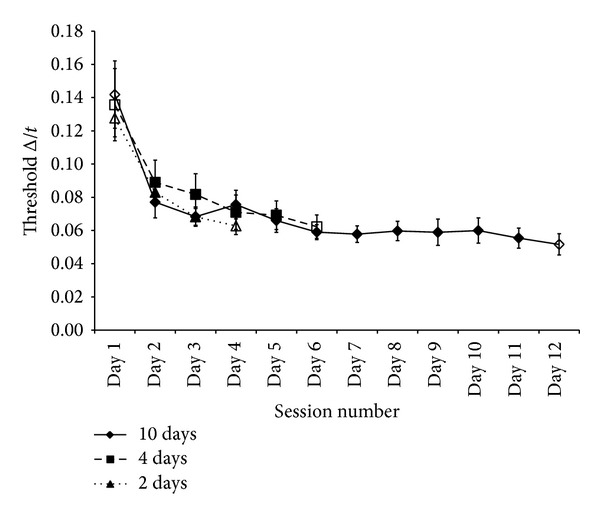
Learning curves showing mean temporal-duration discrimination (Δ*t*/*t*  for 79% correct performance) on the trained standard interval (90 ms/1 kHz,  −85 dB). Shapes on the lines represent experimental groups defined by number of training days (♦ = 10 d, ■ = 4 d, ▲ = 2 d) with filled symbols showing pre- and posttest sessions. All other “days” are training days. Error bars indicate ±1 SEM.

Figure 3 summarises the results for specific learning of the standard interval (90 ms, 1 kHz, −85 dB) over time. At pretest there was no significant difference in the baseline scores for the three groups (*F*(2,23) = 0.147, *P* = 0.864, *r* = 0.08), and so levels of improvement from pre- to posttest can be attributed to task duration. All three groups improved over time from pretest to posttest (mean as a Weber fraction Δ*t*/*t* = 0.076) as would be expected. A 2 time (pre- and posttest) × 3 group (2 d, 4 d, 10 d) ANOVA with time as a repeated measure showed this to be highly significant (*F*(1,21) = 52.392, *P* < 0.0001, *r* = 0.84). However, the amount of time training had little effect with no “time × group” interaction (*F*(2,21) = 0.485, *P* = 0.623, *r*  = 0.15) as all groups improved with equal magnitude. Improvement over the first 3 sessions (pretest to training day 2) displayed a similar trend in that all groups improved over this time (*F*(2,42) = 43.663, *P* < 0.0001, *r* = 0.71) and again at a similar magnitude (*F*(4,42) = 0.508, *P* = 0.730, *r* = 0.11). Due to the possibility of a disparate number of blocks in the pretest (5) and training days (9) influencing the means, a 2-time (training days 1 and 2) × 3 group (2 d, 4 d, and 10 d) ANOVA with repeated measures on time was conducted. All groups improved over these 2 days (*F*(1,21) = 11.296, *P* = 0.003, *r* = 0.59) with no “time × group” interaction (*F*(2,21) = 0.58,   *P* = 0.569, *r* = 0.16). A final comparison in specific learning was to evaluate whether this improvement continued after the 2nd day of training. A 5-time (pretest, training days 1 to 4) × 2 group (4 d, 10 d) ANOVA with time as repeated measures showed that this specific learning continued over time (*F*(4,56) = 19.256, *P* < 0.0001, *r* = 0.51) with equal amounts of learning for both groups (*F*(4,56) = 0.459, *P* = 0.766, *r* = 0.09). Again to account for different block numbers, the same analysis was conducted for these two groups from training days 1 to 4 with an improvement over time, albeit smaller than from pretest (*F*(3,42) = 2.868, *P* = 0.048, *r* = 0.25) with no group interaction (*F*(3,42) = 1.225,   *P* = 0.312, *r* = 0.17).

The results from the specific learning aspect of the experiment indicate that all groups improved over time, indicated by a lowering in discrimination thresholds from pre- to posttest. Division into the experimental group was used to show whether this learning over time was consistent. As there was no significant difference between the three groups, the implications are that the rate of specific learning is not dependent on the total amount of training and that the magnitude is equal across groups. Temporally, the largest amount of improvement was displayed over the first 3 or 4 sessions with any further learning over time at a lower magnitude (for all groups). This suggests that whilst initial specific learning is rapid, further learning can be viewed as fine tuning.

### 3.2. Generalization

**Figure 4:**
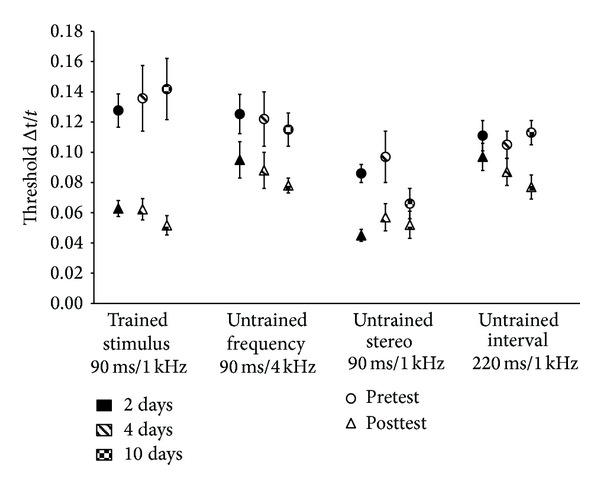
Mean temporal duration discrimination thresholds (Δ*t*/*t* for 79% correct performance) for the trained interval (90 ms/1 kHz), untrained frequency (90 ms/4 kHz), untrained stereo (90 ms/1 kHz), and untrained interval (220 ms/1 kHz). Circles represent pretest scores with triangles showing the posttest scores. Experimental groups are differentiated by colours (2 d training, filled, 4 d, diagonal stripes, and 10 d, chequered). Error bars indicate ±1 SEM. All groups improved on each condition from pre- to posttest, but there was only a significant group difference for generalization for the untrained duration condition where learning transfer was only found for the 10-day condition.

Figure 4 summarises the results for pre- and posttest scores for the trained standard duration (90 ms, 1 kHz, −85 dB), and untrained frequency (90 ms, 4 kHz, −85 dB), stereo (90 ms, 1 kHz, −85 dB stereo), and interval (220 ms, 1 kHz, −85 dB) conditions. Whilst the former tests for the specific learning were described in Section 3.1, the latter three indicate generalized learning. At baseline there were no group differences for frequency (*F*(2,23) = 0.150, *P* = 0.861, *r* = 0.08), stereo (*F*(2,23) = 1.638, *P* = 0.218, *r* = 0.26), or duration (*F*(2,23) = 0.204, *P* = 0.817, *r* = 0.19), again implying that group differences in improvement from pre- to posttest was resultant of duration of training on the standard interval.

### 3.3. Frequency

The alternate frequency condition tested generalization to a spectral feature of the algorithm but retained the same temporal features as the trained standard. A 2-time (pre- and posttest) × 3 group (2 d,4 d, 10 d) ANOVA with time as a repeated measure showed that all groups improved over time from pre- to posttest (*F*(1,21) = 29.712, *P* < 0.0001, *r* = 0.77) with a total mean reduction in discrimination threshold of (*M* = 0.034). However, this was not dependent on group as there was no significant time × group interaction (*F*(2,21) = 0.089, *P* = 0.915, *r* = 0.06). This suggests that generalization to the untrained frequency occurred very early in the time course (2 days) in comparison with the “simple” auditory paradigm (4–10  days). 

### 3.4. Duration

In contrast to the frequency condition the alternate interval condition tested the temporal features of the multimodal signal while retaining the spectral features of the trained stimulus. A 2-time (pre- and posttest) × 3 group (2 d, 4 d, and 10 d) ANOVA with repeated measures on time displayed a highly significant main effect of time (*F*(1,21) = 55.668, *P* < 0.0001, *r* = 0.85) with a mean reduction in discrimination thresholds across the full data set of  *M* = 0.022. In this condition the number of training days on the standard duration did have a significant difference on the amount of learning transfer with a significant time × group interaction (*F*(2,21) = 5.240, *P* = 0.014, *r* = 0.45). Contrasting the three groups to show where on the time course this generalization occurred showed that there was no difference between 2- and 4-day groups (*F*(1,14) = 0.600, *P* = 0.452, *r* = 0.20) but a highly significant difference between 10 and 2 days of training (*F*(1,14) = 8.028, *P* = 0.013, *r* = 0.60). It appeared therefore that generalization occurred after 2 days of training. It seems highly likely that this generalization occurred later in the time course as comparison of the 10- and 4-day groups was borderline significant (*F*(1,14) = 4.424, *P* = 0.054, *r* = 0.49). To test if group composition influenced this contrast, both listener age and gender were entered into a 2 × 3 ANCOVA to account for possible individual differences. Whilst age showed no influence (*F*(1,14) = 4.466, *P* = 0.054, *r* = 0.49), there was a significant time × group interaction with gender as the covariate (*F*(1,13) = 5.250, *P* = 0.039, *r* = 0.54). Thus generalization to the alternate temporal interval condition likely occurred somewhere between 4 and 10 days of training on the standard. This is in contrast to training with simple stimuli where no generalization to the untrained interval was found after 10 days of training.

### 3.5. Stereo

In the stereo condition a comparison was made between hearing the signal in stereo, where the *x*-axis is represented by both a time scan and stereo pan, and the monaural condition of the trained interval where the horizontal axis is represented by just the time scan. We hypothesised that the combination of both time and stereo pan would result in a more complex signal than the time scan alone as it requires the processing of two bits of information to elicit the same result. While there were no group differences at baseline there was a significant difference between the stereo and mono- (standard) conditions that contained the same frequency and temporal features (*t*(23) = 6.188, *P* < 0.0001, *r* = 0.79). Although this could convey an advantage for the stereo input over the mono input it must be taken into consideration that due to presentation order at pretest, each listener will have partaken in at least 300 trials at the standard interval (mono) before the stereo condition. With regard to groups differences in the generalization to stereo stimuli a 2-time (pre- and posttest) × 3 group (2 d, 4 d, and 10 d) ANOVA with time as a repeated measure was conducted. As with all the other conditions there was a main effect of time (*M* = 0.032)(*F*(1,21) = 22.841, *P* < 0.0001, *r* = 0.72) in that all listeners improved discrimination thresholds from pre- to posttests irrespective of number of days of training on the standard stimulus. The number of days of training did not have a significant effect on the magnitude of improvement (*F*(2,21) = 1.740, *P* = 0.200, *r* = 0.10).

The results from the generalization section of the paradigm show that all groups improved on all conditions from pre- to posttest. Group comparisons however showed that, unlike Wright et al. [11], training on the specific duration significantly increased the magnitude of learning on the alternate temporal duration. In this condition generalization occurred in the latter stages of the time course with only the 10- day training group showing this significant improvement. Improvement on the frequency condition was rapid within the first few sessions of training, whilst the use of the stereo input conveyed an advantage over the monaural input at both pretest and posttest. Indeed the posttest means for this condition were lower than those for the trained standard condition, implying an overall benefit of utilising the stereo input.

## 4. Long Term Maintenance of Perceptual Learning

Experiment 2 was conducted to ascertain whether the perceptual learning achieved in experiment 1 was maintained over time. Listeners from the 10-day training group were invited back to take part in another test phase session. This session was identical to the pre- and posttest sessions of the original experiment, that is, 4 conditions, 5 blocks per condition. Of the original group of listeners, seven of eight returned for testing. This group was further partitioned based on the time that had elapsed since finishing the experiment 1 posttest. For 5 listeners this time was equal to 6 months whilst for the remaining two, 3 months. Both the experimental setup and location were exactly the same as experiment 1.

### 4.1. Long Term Learning

**Figure 5:**
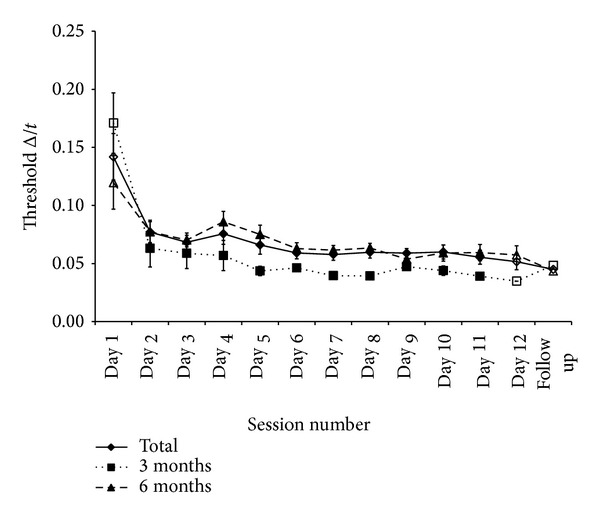
Learning curves showing mean temporal-interval discrimination thresholds (Δ*t*/*t*  for 79% correct performance) on the trained standard interval (90 ms, 1 kHz, −85 dB). The filled line represents the full data set for this experiment with the dotted line showing the curve for the “3 months since experiment 1 posttest” group and the dashed line for the “6 months from experiment 1 posttest” group. Filled symbols represent test phases (pre-test, posttest, follow-up) with empty symbols being training days. Whilst not displayed to scale, the first 12 sessions all took place within 3 weeks of the pretest session with the “follow- up” session undertaken either 3 or 6 months after the posttest session. Error bars indicate ±1 SEM.

Figure 5 summarises the results for the specific learning on the trained interval (90 ms/1 kHz,  −85 dB) after either 3 or 6 months from posttest. Collectively there was a significant improvement from pretest to follow-up session shown by a two time (pretest, follow-up) × two group (6 months, 3 months) ANOVA, with time as a repeated measure (*F*(1,5) = 19.482,   *P* = 0.007, *r* = 0.89). However, as there was no significant time × group interaction (*F*(1,5) = 1.069, *P* = 0.349, *r* = 0.42) the duration from completion of experiment 1 had no significant influence on the maintenance of the perceptual learning. While group differences were not significant the average improvement for the 3-months groups (*M* = 0.122) was larger than the 6-months groups (*M* = 0.076).

When considering difference from posttest to follow-up for the trained interval there was neither a main effect of time (*F*(1,5) = 0.000,   *P* = 0.986) or group × time interaction (*F*(1,5) = 3.005, *P* = 0.144,   *r* = 0.61). The mean scores showed that while there was a small decline in scores from posttest to follow-up for the 6-months groups (*M* = −0.009), the 3-months groups actually improved between these two points on the time course (*M* = 0.002).

### 4.2. Long Term Generalization

**Figure 6:**
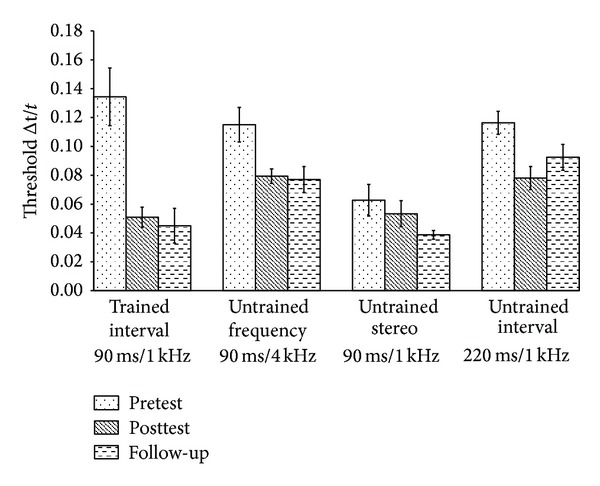
Discrimination thresholds (Δ*t*/*t*  for 79% correct performance) on the trained standard duration (90 ms,1 kHz,−85 dB) and untrained frequency (90 ms, 4 kHz, −85 dB), stereo (90 ms, 1 kHz, −85 dB), and duration (220 ms, 1 kHz, −85 dB) conditions. Light cheques indicate pretest scores and dark grey bars posttest scores from experiment 1. Dark chequed bars indicate scores from the follow-up study, experiment 2. Error bars indicate ±1 SEM.

Figure 6 summarises the results for generalized learning to the untrained frequency (90 ms, 4 kHz, −85 dB), stereo (90 ms,   1 kHz,  −85 dB), and duration (220 ms,   1 kHz,  −85 dB) conditions from both pre- and posttests. Considering frequency first, a two-time (pretest, follow-up) × two group (6 months, 3 months) ANOVA with repeated measures showed that there was a borderline significant main effect of time for the full data set (*F*(1,5) = 6.486, *P* = 0.051, *r* = 0.75) and that this improvement was not dependent on group (*F*(1,5) = 0.259, *P* = 0.632, *r* = 0.22). All listeners showed lower discrimination thresholds at the long term follow-up than at pretest, implying that improvements due to the training on the standard interval between pre- and posttests were at least maintained if not improved over durations of 3 and 6 months even without any additional training. During experiment 1, there was a considerable improvement between pre- and posttests for the alternate frequency condition, so the carry over in performance improvement to follow-up is not surprising. The results from experiment 1 show that an apparent ceiling level threshold is reached, displaying a maximum benefit of training that would not be exceeded with additional sessions. Therefore when comparing posttest (where most listeners had attained this definitive threshold) and follow-up, we would not expect any further improvement. Indeed when contrasting these two points on the time course for the frequency condition, there was no significant main effect of time (*F*(1,5) = 0.369, *P* = 0.570, *r* = 0.26) or time × group interaction (*F*(1,5) = 4.691, *P* = 0.083, *r* = 0.70). However, while the 3-months group showed a diminishment in the amount of learning transfer (*M* = −0.019), the 6-months group actually showed a small level of improvement at follow-up compared to posttest (*M* = 0.002); at the very least this suggests that the subjects maintained the level of performance achieved at the end of the training 6 months earlier.

A similar counter intuitive result was also found when looking at the stereo condition. From pretest to follow-up, while there was no significant main effect of time (*F*(1,5) = 4.106, *P* = 0.099, *r* = 0.07) or time × group interaction (*F*(1,5) = 0.018, *P* = 0.898, *r* = 0.06), there was a small mean improvement for both the 6-months (*M* = 0.024) and the 3-months conditions (*M* = 0.026). When contrasting the posttest to follow-up again there was no significant effects of time (*F*(1,5) = 0.114, *P* = 0.749, *r* = 0.15) or time × group interaction (*F*(1,5) = 5.764, *P* = 0.62, *r* = 0.73). However, on looking at the means, the 6-months group showed a lower discrimination threshold at follow-up than at posttest with an improvement of 0.029. This was not evident for the 3-months condition where there was a diminishment at follow-up of −0.022. 

When looking at the follow-up data for the alternate untrained duration (220 ms), there was an overall significant improvement from pretest to follow-up (*F*(1,5) = 10.405, *P* = 0.023, *r* = 0.82), and although this was not dependent on group (*F*(1,5) = 2.406, *P* = 0.182, *r* = 0.57), the mean scores showed that the performance improvement was larger for the 3-months (*M* = 0.044) than 6-months condition (*M* = 0.015). However, as the initial baseline thresholds were considerably higher (worse) for the 3-months group, the implications are that the main improvement was within the training days rather than in the “break” posttest. From posttest to follow-up, there was no main effect of time (*F*(1,5) = 2.280, *P* = 0.191, *r* = 0.56) or time × group interaction (*F*(1,5) = 0.961, *P* = 0.372, *r* = 0.40), and whilst the means showed that both groups performed worse on the alternate duration after the respective break the diminishment of performance was minimal and therefore the perceptual learning had been maintained over three and six months. 

## 5. Discussion

The purpose of this study was to evaluate whether the perceptual learning of complex auditory stimuli might result in greater and longer lasting generalization than previously reported in the literature. First, similar specific learning results were found despite the increased complexity of the stimuli. Second, we discovered the first instance, to our knowledge, of generalization to a novel temporal duration within stimulus type in contrast to the prior temporal generalization found by Lapid et al. [14] where the generalization was across stimulus types (empty to filled). We deem this within stimulus temporal generalization important as this is ecologically valid to everyday processes such as speech which predominantly consists of filled soundscapes. Third we also assessed for the first time whether the improvements brought about through auditory perceptual learning could be maintained over a long delay period of three to six months, and indeed we found that the benefits of specific and generalized learning were retained. 

As with the results in the foundational work in interval discrimination by Wright and colleagues, specific learning of the trained duration (90 ms, 1 kHz) occurred early in the time course with a statistically significant improvement shown by the first test day (after two 540 trial training days). Indeed there was a significant improvement after only 1 training day, but due to a disparate number of blocks between test and training phases this should be approached with caution. Generalization to untrained conditions occurred later in the time course implying different neural processes for specific and generalized learning. A significant improvement for the untrained frequency (90 ms, 4 kHz) was found somewhere between 2 and 4 days of training, more rapidly than in the simple unisensory paradigm, with the novel findings of generalization to the untrained duration (220 ms, 1 kHz) occurring later in the time course (between 4 and 10 days). We can therefore draw similar conclusions to Wright in that generalization to novel stimuli requires a distinct amount of training. The use of complex stimuli extends the previous work in that it not only appears to decrease the amount of training required for generalization to occur (frequency) but also increases the breadth of generalization (duration) facilitated by training on a standard interval. Utilising complex and multisensory stimuli also draws comparisons with speech perception, where complex signals and auditory-visual multisensory processes are the norm [30, 31].

In postulating an explanation for the novel results found in the complex stimuli learning paradigm, here we consider theories of perceptual learning, the possible neural networks involved, whether the complex composition of the stimuli would facilitate the use of alternate networks, and finally if the stimuli are actually being processed solely as auditory signals.

While Wright and colleagues [11, 15, 16] speculated that, due to different positions on the time course, specific and generalized learning may utilise different or modified neural circuitry, this may be further influenced by the neural networks that facilitate spectral and temporal processing and how they are integrated in the multisensory signal. Auditory processing is assumed to be analogous to the visual system in that two functional pathways are utilised to process “what” and “where” information [32]. For the latter the posterodorsal pathway from the primary auditory cortex (A1) through the posterior temporal lobe and posterior parietal lobe to the dorsolateral frontal lobe has been proposed for spatial processing with the anteroventral pathway from A1 through anterior temporal lobe to inferior frontal lobe coding the “what” features of the signal. 

Whether the signal is being processed as unisensory or multisensory the reverse hierarchy theory of perceptual learning provides a theoretical explanation for the results found utilising complex stimuli [18]. Primary to this theory is that perceptual learning can happen at any level of processing, and it is the complexity and difficulty of the task which guides the level at which the processing occurs. Difficult tasks, where more specific discrimination is required, focus attentional resources to primary sensory areas. However, if the task can be accomplished utilising more general object features the processing drives attention to higher levels. The reverse hierarchy theory can be applied to both unisensory and multisensory learning using similar mechanisms. Modality specific unisensory learning is supported by either low-level auditory areas for specific learning or high-level auditory areas for general learning [17, 18]. Learning utilising multisensory stimuli can lead to correlated activity in higher-level multisensory areas [33] or learning can progress from primary sensory areas to higher-level multisensory areas under complex unisensory stimulation. Activity may then cascade back down the hierarchy such that generalization across modalities occurs when these higher-level multisensory areas are implicated in learning either unisensory or multisensory tasks [34].

This naturally raises the question as to whether the novel generalization found to the alternate temporal interval in the multisensory paradigm is due to the spatiotemporal composition of the sonified image or can be attributed purely to the complexity of the signal. Future research could evaluate this by creating auditory stimulus sets which incorporate a number of frequency bands superimposed over each other to create a complex, but still unisensory, signal. If generalization to the alternate temporal duration is not found in this complex signal, then the implications are that it is the multisensory nature of the signal that is driving temporal generalization rather than complexity per se.

A final consideration concerns the results from Experiment  2. To our knowledge this is the first evaluation of such long term benefits of perceptual learning in the auditory or multisensory domains, and it provides invaluable information for developing long term training protocols. Performance in all conditions was not only superior to results for the pretest phase but, alternate duration aside, also superior to the posttest phase. This implies that the specific and generalized learning attained through training is maintained over considerable lengths of time even without additional training. The results from posttest to follow-up, that is, participants continue to improve over periods of no training, is somewhat counterintuitive. We have to be wary of stating that this is an experimental effect due to the low number of listeners in the 3- months group; however, we can theorise why these incongruous results occur. In structured interviews with long term users of The vOICe, it was reported that one user experienced vOICe like visual percepts evoked by auditory stimulation even when not using the device. These were elicited by environmental sounds that were vOICe-like in composition but not multisensory in nature [35]. It may therefore be possible that exposure to such sounds is strengthening neural networks instigated or unmasked through device use. If this is so, then the 6-months follow-up group may have been exposed to more of these sounds than the 3-months group, and therefore the learning network is further strengthened, hence the greater improvement for the longer absence. Future experiments could test for this by providing post-training listeners with complex unisensory sound stimuli in a nonstructured setting between posttest and follow-up.

Whether it is the complex nature or the multisensory aspects of the signal that are driving generalization, there are implications on how training protocols are structured for both unisensory learning and training on sensory substitution. Training paradigms in sensory substitution generally utilise simple objects and build complexity over time. Whilst this is a logical approach, if complexity is facilitating generalization, then it may be more advantageous to initially raise the level of object complexity for an improved long term gain. The learning is maintained over periods without explicit training also allows for the continuation of a training regime at its cutoff point if an enforced break has occurred. Advantages in learning sensory substitution may also be conveyed by incorporating complex unisensory auditory tasks into the training protocols. Indeed this could also be bidirectional in utilising sensory substitution in auditory training such as speech therapy.
